# Remote Ischemic Preconditioning Neither Improves Survival nor Reduces Myocardial or Kidney Injury in Patients Undergoing Transcatheter Aortic Valve Implantation (TAVI)

**DOI:** 10.3390/jcm9010160

**Published:** 2020-01-07

**Authors:** Mandy Flechsig, Tobias F. Ruf, Willi Troeger, Stephan Wiedemann, Silvio Quick, Karim Ibrahim, Christian Pfluecke, Akram Youssef, Krunoslav M. Sveric, Robert Winzer, Frank R. Heinzel, Axel Linke, Ruth H. Strasser, Kun Zhang, Felix M. Heidrich

**Affiliations:** 1 Department of Internal Medicine and Cardiology, Herzzentrum Dresden at Technische Universität Dresden, 01307 Dresden, Germany; 2 Center for Cardiology, Cardiology I, University Medical Center Mainz, 55131 Mainz, Germany; 3 Department of Internal Medicine and Cardiology, HELIOS Klinikum Pirna, 01796 Pirna, Germany; 4 Department of Cardiology, Klinikum Chemnitz, Technische Universität Dresden, 09116 Chemnitz, Germany; 5 Department of Radiology, Universitätsklinikum Dresden, 01307 Dresden, Germany; 6 Department of Internal Medicine and Cardiology, Charité—Universitätsmedizin Berlin, Campus Virchow-Klinikum, 13353 Berlin, Germany; 7 Medical Faculty, Technische Universität Dresden, 01069 Dresden, Germany; 8 Berlin Health Institute, 10178 Berlin, Germany

**Keywords:** aortic valve stenosis, transcatheter aortic valve replacement, ischemic preconditioning, bioprosthesis

## Abstract

Background: Peri-interventional myocardial injury occurs frequently during transcatheter aortic valve implantation (TAVI). We assessed the effect of remote ischemic preconditioning (RIPC) on myocardial injury, acute kidney injury (AKIN) and 6-month mortality in patients undergoing TAVI. Methods: We performed a prospective single-center controlled trial. Sixty-six patients treated with RIPC prior to TAVI were enrolled in the study and were matched to a control group by propensity-score. RIPC was applied to the upper extremity using a conventional tourniquet. Myocardial injury was assessed using high-sensitive troponin-T (hsTnT), and kidney injury was assessed using serum creatinine levels. Data were compared with the Wilcoxon-Rank and McNemar tests. Mortality was analysed with the log-rank test. Results: TAVI led to a significant rise of hsTnT across all patients (*p* < 0.001). No significant inter-group difference in maximum troponin release or areas-under-the-curve was detected. Medtronic CoreValve and Edwards Sapien valves showed similar peri-interventional troponin kinetics and patients receiving neither valve did benefit from RIPC. AKIN occurred in one RIPC patient and four non-RIPC patients (*p* = 0.250). No significant difference in 6-month mortality was observed. No adverse events related to RIPC were recorded. Conclusion: Our data do not show a beneficial role of RIPC in TAVI patients for cardio- or renoprotection, or improved survival.

## 1. Introduction

Physicians are still seeking a tool that can improve the outcome of patients with myocardial injury. In this regard, remote ischemic preconditioning (RIPC) has attracted broad awareness lately. In RIPC, brief, reversible episodes of ischemia followed by reperfusion are applied in a peripheral tissue or organ. The peripheral stimulus can be chemical, electrical or mechanical and renders protective effects on the heart or another distant organ through neuronal and humoral signaling [1]. Mechanical RIPC by repeated inflation and deflation of a blood pressure cuff is a non-invasive and inexpensive method, which is easily applicable in the clinical routine. Inconsistent results have been published to date concerning the cardioprotective value of RIPC. In patients suffering from myocardial infarction who were treated with percutaneous coronary intervention (PCI), decreased infarct sizes and reduced mortality were reported [2,3,4]. In contrast to promising results in smaller studies [5,6], large surgical trials did not confirm a benefit of RIPC in patients undergoing heart surgery [7,8].

In patients with symptomatic severe aortic stenosis, transcatheter aortic valve implantation (TAVI) has evolved from a bail-out procedure in patients unsuitable for cardiac surgery to the method of choice in patients who are high, intermediate, and also recently, low risk for cardiac surgery [9,10,11,12]. However, myocardial injury occurs frequently in patients receiving TAVI, which is associated with worse outcome [13,14]. It can be caused by several conditions [14,15] including (1) micro-embolism into coronaries, (2) hypoperfusion due to rapid pacing, (3) direct mechanical myocardial injury due to balloon dilatation and prosthesis implantation, and (4) possible complications during the procedure. Moreover, acute kidney injury (AKIN) is regularly observed as a complication following TAVI, being partly related to the amount of injected iodine contrast agent during the procedure. The knowledge about RIPC in patients undergoing TAVI is sparse. Until now, only one study by Kahlert et al. [16] has examined the influence of RIPC in TAVI patients. However, it did not provide evidence for a protective effect on the heart, kidney and brain, or an improved outcome.

In this study, we sought to elucidate the effect of RIPC prior to TAVI on acute myocardial and renal injury, as well as on mortality, after six months.

## 2. Materials and Methods

### 2.1. Study Design and Patient Enrolment

**Figure 1:**
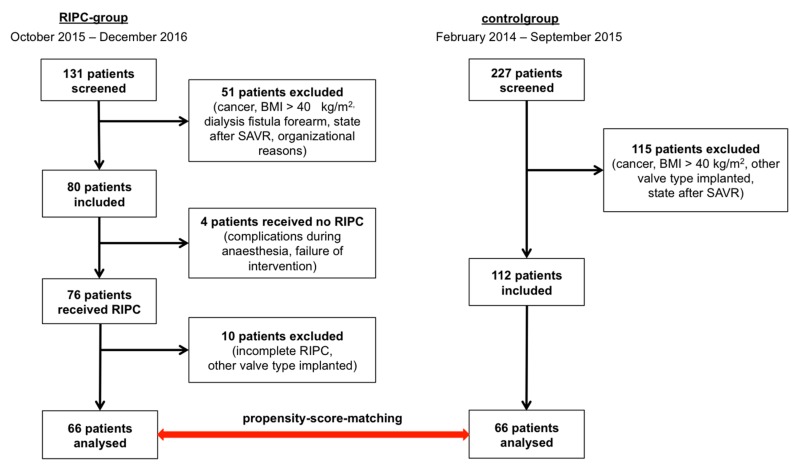
Study design and patient enrolment. Left column shows RIPC-group, right column shows control group. Exclusion criteria were participation in other trials, body mass index (BMI) > 40 kg/m^2^, second intervention because of bioprothesis degeneration (“*valve-in-valve*”), active malignancy with life-expectancy less than 1 year, SIRS or sepsis, cardiogenic shock, dependency on inotropes, peripheral artery disease, thrombosis and chronic renal failure with need for dialysis and dialysis fistula at forearm. BMI = body mass index; SAVR = surgical aortic valve replacement; RIPC = remote ischemic preconditioning.

We performed a prospective single-center controlled trial with patient enrolment from February 2014 to December 2016. The decision for TAVI was made on an individual basis by the interdisciplinary Heart Team in accordance with current guideline recommendations [17]. Exclusion criteria were participation in other trials, second intervention (“*valve-in-valve*”), active malignancy with life-expectancy less than one year, systemic inflammatory response syndrome or sepsis, cardiogenic shock, dependency on inotropes, symptomatic peripheral artery disease, thrombosis and chronic renal failure with need for dialysis (Figure 1). Written informed consent was obtained from all patients prior to TAVI. Data on control group patients (non-RIPC group), who received TAVI between February 2014 and April 2015, were acquired retrospectively. The study was approved by the local ethics committee (reference number #73032013).

### 2.2. Procedure—TAVI and RIPC

**Figure 2:**
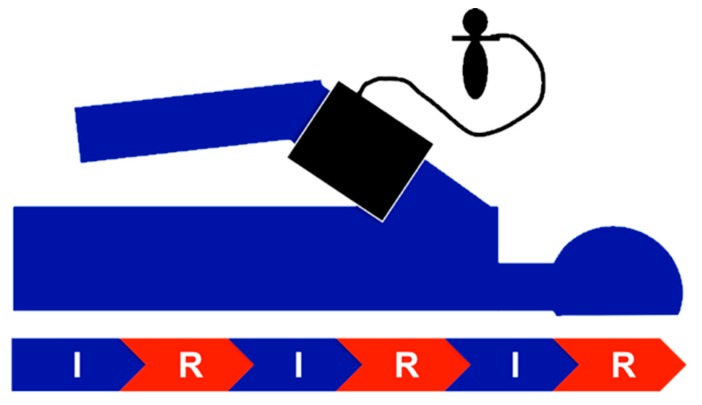
RIPC scheme. Three cycles of ischemia (I) and reperfusion (R) of 5 min each, respectively, were applied, resulting in a total duration of RIPC of 30 min. Efficacy of RIPC was assessed clinically (pulselessness, acrocyanosis, reactive hyperemia). The blood pressure cuff was regularly applied to the right upper arm.

Aortic valves were implanted through the transfemoral approach and under general anesthesia as previously described [9]. Application of RIPC started with the induction of anesthesia. We performed three cycles of ischemia for five minutes followed by reperfusion for five minutes (Figure 2). To induce ischemia, the cuff of a standard blood-pressure-manometer (Boso, Jungingen, Germany) was inflated 20–30 mmHg above the systolic arterial pressure. Efficacy was assessed clinically by pulselessness of radial artery and acrocyanosis followed by reactive hyperemia. The time interval between the end of the RIPC procedure and the start of the TAVI intervention was less than 30 min. Patients of each group received either a self-expandable CoreValve Evolut R (Medtronic, Minneapolis, Minnesota, USA) (n = 44) or a balloon-expandable Sapien XT/Sapien 3 (Edwards Lifesciences Inc., Irvine, CA, USA) (n = 22) valve. We used Imeron 350 (Bracco S.p.A., Milan, Italy) as contrast agent.

### 2.3. Blood Sampling and Analysis

**Figure 3:**
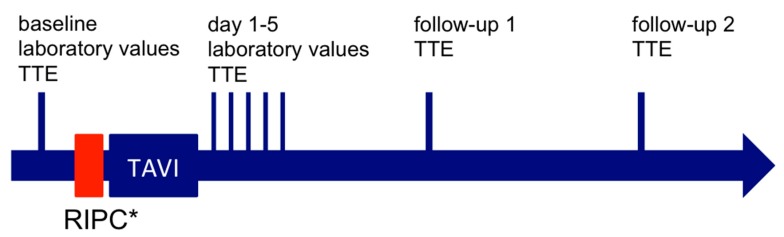
Timeline of events. * RIPC group only. RIPC = remote ischemic preconditioning; TAVI = transcatheter aortic valve implantation; TTE = transthoracic echocardiography.

Venous blood samples were collected at hospitalization and routinely the first five days after the TAVI procedure each morning at rest (Figure 3). Parameters were measured with Cobas 6000 (Roche, Rotkreuz, Switzerland). High-sensitive troponin-T (hsTnT) was measured by immunoassay in a sandwich-technique (cut-off 14 ng/L). Concentrations of creatinine and urea were determined photometrically. Glomerular filtration rate was calculated using the CKD-EPI-formula.

### 2.4. Endpoints

The primary study endpoint was the characterization of myocardial injury as reflected by hsTnT kinetics in both groups (RIPC, non-RIPC). We calculated the respective area under the curve (AUC) by the trapezoid method. Myocardial injury was assessed according to the current Valve Academic Research Consortium (VARC)-2 recommendations [18]. Patients with baseline hsTnT below the upper limit of normal (ULN) (14 ng/L), which rose above the ULN after the TAVI procedure, were classified as having myocardial injury, as were patients with baseline hsTnT concentrations above the ULN and an additional increase of 20% [19]. Secondary endpoints were events of acute kidney injury (AKIN) according to KDIGO, echocardiographic changes and 6-month mortality. Moreover, other peri-procedural complications were also recorded. Transthoracic echocardiography was performed prior to the intervention, post-procedure and at follow up visits after 3 and 6 months, respectively. Patients unable to participate in our institution’s follow-up program were instead followed up by their local cardiologists.

### 2.5. Statistical Analyses

RIPC group patients were matched to control group patients by propensity score. The method used was nearest-neighbour by MatchIt-package [20] using the statistics software package “R” (The R Foundation for Statistical Computing, Vienna, Austria). Patients were matched according to the type of valve implanted. Possible influencing factors of troponin elevation were considered with the variables sex, pre-procedural ejection fraction, frequency of relevant coronary artery disease with more than 50% stenosis, frequency of peri-procedural pacing runs, volume of contrast agent used during the procedure and pre-procedural creatinine concentration as a marker of pre-existing kidney injury. To obtain these variables as matching covariates, we compared the baseline variables of both groups with standardized differences [21]. All tests were run by Statistical Package for Social Sciences, version 23 (SPSS Inc., IBM, Armonk, NY, USA). Data are presented as means and inter-quartile-ratios (IQRs), unless stated otherwise. *p*-values < 0.05 were considered statistically significant. Normally distributed continuous data was compared by paired-samples t-test, and non-normally distributed data by the Wilcoxon signed-rank test. Distribution of normality was assessed by the Shapiro–Wilk and Kolmogorov–Smirnov test. Dichotomous data was analyzed with McNemar’s test and the survival analysis was done by the Kaplan–Meier procedure and Log Rank test. For missing hsTnT concentrations, an imputation method was used by a linear mixed model as quadratic development over the baseline troponin concentrations [22].

## 3. Results

In total, 358 patients were screened for eligibility to participate in our study, i.e. 131 RIPC and 227 non-RIPC patients (Figure 1). Patients not meeting the inclusion criteria were excluded. Eventually, 66 RIPC patients and 66 matched control subjects were available for statistical analysis. One patient was lost to follow up in the control group.

The baseline characteristics of the unmatched cohorts are given in Table S1, as are the standardized differences of the matching variables in Table S2. Following propensity matching, both groups (RIPC and matched non-RIPC groups) showed no statistically significant differences in baseline and procedural parameters (Table 1). We observed no adverse events related to RIPC.

### 3.1. Myocardial Injury

**Figure 4:**
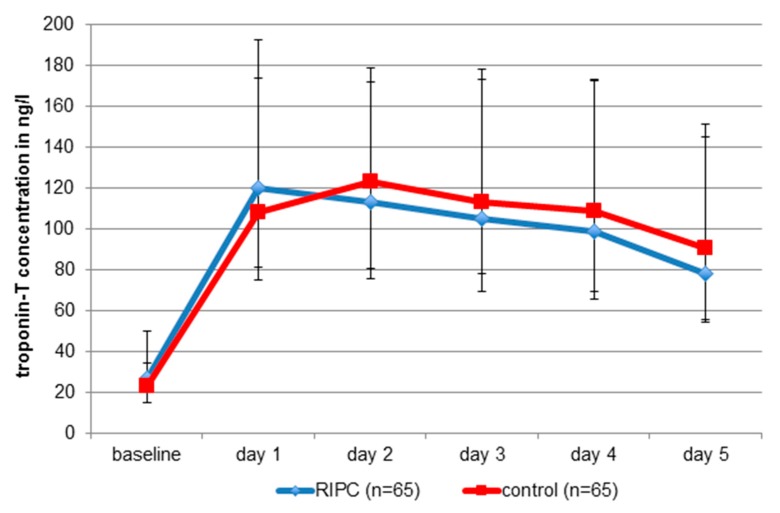
Absolute hsTnT concentrations of the RIPC group and matched control group patients. No statistically significant differences between groups with respect to maxima and area under the curves. Values are median (IQR). RIPC = remote ischemic preconditioning.

Myocardial injury was common, occurring in 63 cases of the RIPC group (97%) and in 64 cases of the control patients (99%). Increased baseline hsTnT levels occurred in 78% of all patients. Peri-procedural myocardial infarction was not registered in any of the two groups (Table 2). Following TAVI, there was a significant rise in cardiac troponin across all patients (*p* < 0.001), peaking day 1 in the RIPC group (120 ng/L [81.5–192.5]) and day 2 in the non-RIPC group (123 ng/L [80.5–172.0]) (Figure 4). There were no statistically significant inter-group differences in the maximum troponin release (Figure 4) or the AUC at five days (RIPC: 485.5 ng/l/d (352.3–810.0); control: 502.0 ng/l/d (IQR 359.3–820.5); *p* = 0.60). One patient was excluded from analysis due to troponin rise after severe pulmonary embolism.

**Figure 5:**
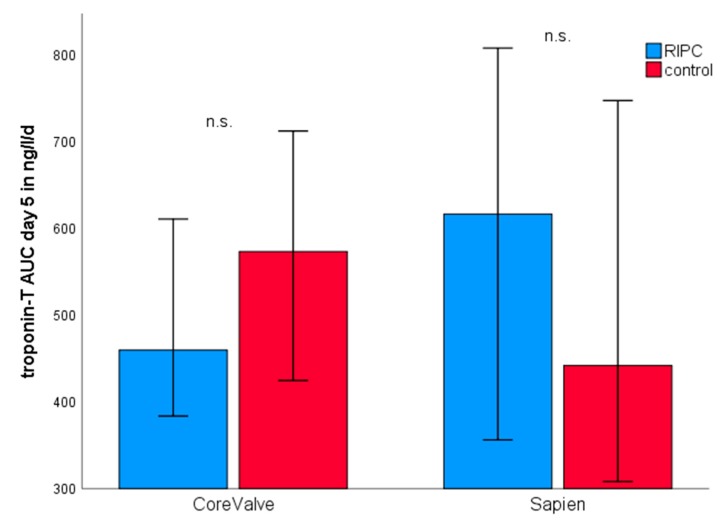
Troponin release according to the type of bioprosthesis. Blue columns show RIPC group, red columns show control group. There was no statistically significant difference between RIPC and non-RIPC patients for either bioprothesis used. Values are median and 95%-CI as error bars. RIPC = remote ischemic preconditioning; AUC = area under the curve.

Regarding the type of valve implanted, Medtronic CoreValve and Edwards Sapien XT/3 valves showed similar peri-interventional hsTnT maxima and kinetics (CoreValve 135.0 ng/L (93.3–208.8), n = 86; Sapien XT/3 127.5 ng/L (77.0–198.0), n = 44; *p =* 0.665). RIPC had no statistically significant impact in combination with either bioprosthesis (Figure 5).

### 3.2. Kidney Injury

Creatinine peak, creatinine AUC at five days and change of GFR did not differ significantly between both groups (Table 3). AKIN occurred in one RIPC patient and four non-RIPC patients (*p =* 0.250) following TAVI. None of the patients needed dialysis.

### 3.3. Echocardiography

Echocardiographic parameters differed neither before nor after TAVI between the two groups (Table S3). In both groups, aortic stenosis was successfully treated, as reflected by highly significant decreases of Vmax, dpmax and dpmean (*p* < 0.001). Left ventricular ejection fraction and aortic regurgitation were similar in both groups post-intervention.

### 3.4. Mortality

**Figure 6:**
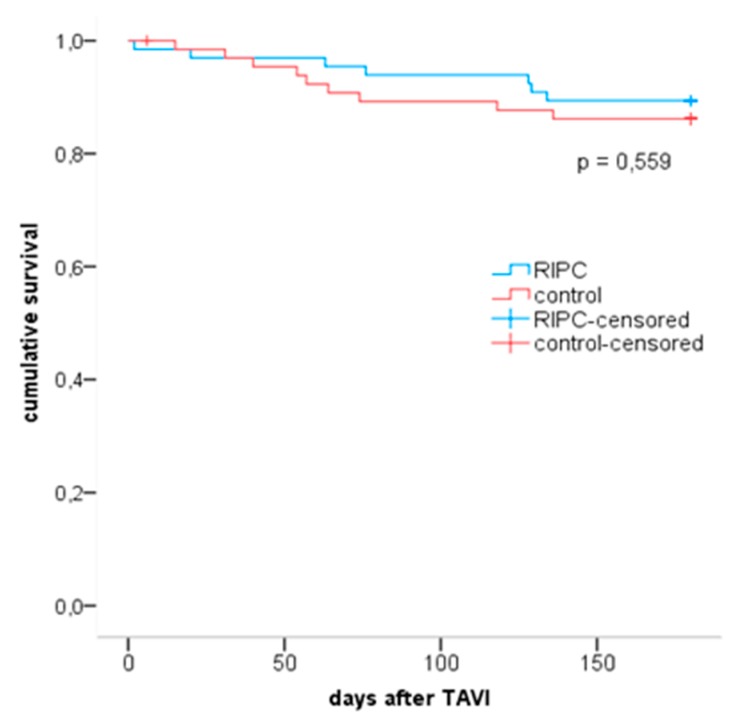
Kaplan–Meier curves for RIPC and matched control group. Blue line shows RIPC group, red line shows control-group. Numbers at risk: time point 0—n = 66 for RIPC and controls; time point 6 months—n = 59 [RIPC], n = 57 [controls]. RIPC = remote ischemic preconditioning; TAVI = transcatheter aortic valve implantation.

All-cause mortality did not significantly differ at 30 days, 3 months and 6 months after TAVI (Figure 6). Within 6 months, 7 RIPC patients and 9 control patients died (*p* = 0.559). All deceased patients (n = 16; 12%) showed a significantly higher hsTnT concentration at baseline compared to all other patients (42.5 ng/L (72%) for deceased patients, 23.0 ng/L (23%) for patients who survived; *p* = 0.012).

### 3.5. Complications According to VARC-2

Atrial flutter/fibrillation, atrioventricular and branch blocks were common after TAVI in both groups. New pacemaker implantations were not significantly different. Patients from the RIPC group showed three cases of valve thromboembolism, making implantation of another bioprosthesis (valve-in-valve-procedure) or conversion to open heart surgery necessary. Other complication rates were not statistically different between both groups (Table 2).

## 4. Discussion

The objective of the current study was to explore the effects of RIPC on myocardial injury, kidney injury and mortality in patients undergoing TAVI for severe aortic stenosis. Our data show a significant rise of hsTnT across all patients following TAVI, indicating myocardial injury. However, myocardial injury was not mitigated by RIPC across all valves implanted, and when considering Medtronic CoreValve and Edwards Sapien bioprostheses separately. Patients receiving RIPC prior to TAVI did not benefit in terms of kidney injury or failure. Neither could we show a benefit of RIPC in TAVI patients with respect to mortality after one, three and six months. The RIPC procedure, however, was well tolerated without any related adverse events.

### 4.1. Baseline Characteristics

With respect to baseline criteria, our study population is comparable to other studies [12,23]. Up to this point the effect of RIPC in TAVI patients was investigated only by Kahlert et al., 2017 [16]. In their and this present study, RIPC patients did not benefit in terms of cardioprotection, renoprotection or mortality.

### 4.2. Type of Bioprosthesis

One central part of our study was the investigation of the RIPC effect in different types of transcatheter biological aortic valves. The self-expandable CoreValve bioprosthesis and the balloon-expandable Sapien bioprosthesis are the two main devices in clinical use. The choice of valve type is mainly dictated by the size of the native annulus and the cardiac anatomy. Although device specific complications are evident, there are no differences in clinical outcomes in terms of short- or long-term survival with use of either valve [24]. Due to the use of a CoreValve prosthesis, the incidence of permanent pacemaker implantation was 33% in this study as compared to 8% in the study by Kahlert et al., who only included Edwards prostheses. From today’s point of view, one major limitation of this previous study is the exclusive use of Sapien XT prostheses. Our study did not reveal a valve-specific impact on myocardial injury. Troponin-T-peak and AUC were similar in both groups, which is reasonable as both valve types showed comparable number of implantations, sizes, pacing runs and additional dilatations in the subgroup analysis.

### 4.3. Mortality and Myocardial Injury

For a period of up to 6 months, RIPC did not achieve a significant difference in mortality compared to the control group (n = 7 RIPC group vs. n = 9 control group, *p* = 0.559). Our study participants represented the common patient population undergoing TAVI with an average age of 82 years and multiple co-morbidities. An increased hsTnT baseline is a sign of multimorbidity and a predictive parameter of patient outcomes [25]. The baseline hsTnT was significantly higher in the 16 patients who died compared to the survivors. At present, the only established surrogate parameter to assess the extent of peri-interventional myocardial injury is measurement of troponin and there is evidence for the correlation of higher troponin levels with higher mortality [19,26,27,28]. The majority of patients (98%) in this study suffered myocardial injury following TAVI, confirming previous study results [19,29]. However, this did not influence left ventricular function as echocardiographic evaluation of ejection fraction showed. To clearly differentiate between myocardial injury and infarction, we followed current VARC-2 recommendations [18] and reference levels from other studies [19,29] and defined peri-procedural infarction as an increase of troponin above 15 times of URL within 72 h plus presence of clinical manifestations (ischemia in electrocardiogram, abnormal myocardial motion in echocardiography) in contrast to injury where troponin increase is no more than 20%. No patient in the current study suffered peri-interventional myocardial infarction.

### 4.4. Renoprotection by RIPC

The amount of contrast agent within the TAVI procedure is important for a possible kidney injury. The amount used in the current study population (142 mL RIPC group vs. 137 mL control group) is comparable with numbers from “The German Aortic Valve Registry” (GARY; 27 participating centers, 165 mL contrast agent [30]). With 3.8%, occurrence of renal injury without renal replacement therapy was below average [13].

In two large clinical trials RIPHeart [31] and ERICCA [8], RIPC did not reduce major perioperative adverse cardiac, renal and cerebral events in bypass surgery patients with or without valve replacement. In contrast to that, Zarbock et al. showed that RIPC reduced the rate of acute kidney injury and use of renal replacement therapy in patients undergoing bypass surgery. In contrast to the RIPHeart and ERICCA trials, there were differences in the anesthetic regimen (no propofol) and prior medication (no sulfonylurea), and only patients at a high risk of acute kidney injury were included. Of note, our study showed a small trend towards renal protection (acute kidney injury: n = 1 RIPC group vs. n = 4 control group, *p* = 0.250), but this was not statistically significant. Comparing these previous studies with our present, the following points and differences need to be considered: (1) different cohort (cardiac surgery), (2) preconditioning may be less effective in patients with infarct-remodeled hearts, (3) cardiopulmonary bypass, hypothermia and cardioplegia itself are known to be protective (perhaps further protection is impossible to achieve), and (4) concomitant medications may interfere with remote ischemic preconditioning.

### 4.5. Pharmacological Confounding Factors

RIPC is believed to convey its positive effects on ischemia/reperfusion injury through neural and hormonal pathways. Multiple pharmaceuticals, such as statins, platelet inhibitors and general anaesthesia, including opioids, hypnotics and sedatives, might consequently influence the outcome in a trial of RIPC. For instance, the influence of anesthesia is a matter of debate, particularly the lack of RIPC effect in some studies where propofol was applied [6,7,8]. On this basis, the popular trials ERICCA and RIPHeart received some criticism [32]. An alternative drug that has been used to avoid propofol was midazolam, which however did not unmask an effect of RIPC either [16]. On the other hand, publications also exist that confirm an effect of RIPC despite usage of propofol [5,33,34]. Opposed to that, a variety of medications may possess cardioprotective effects in the sense of pharmacological preconditioning. In summary, the confounding effect of various drugs has not been finally resolved. While we cannot exclude a general effect of pharmaceuticals on the outcome of RIPC, we can safely assume that there were no inter-group differences regarding common cardiac medications and type or dose of anesthesia.

### 4.6. Best Practice of RIPC

There are several studies, animal as well as clinical trials, examining the potential beneficial effect of ischemic preconditioning [1,5,6,35,36,37]. While there is still a lack of evidence if pre, per-, or post-conditioning is superior to one of the others [38,39,40], we decided to perform preconditioning because application of RIPC before the TAVI procedure was most convenient time point to implement into the TAVR routine, assuring the best consistency in performance. While there is still no sufficient proof for the optimal regimen regarding RIPC duration [3,41,42] or number of cycles [43], we took the most commonly used version (3 cycles of 5 min. ischemia) derived from studies in cardiac surgery as well as cardiology [2,4,5,6,44]. It remains unresolved whether expansion of ischemia areas, such as on both arms or both legs, would amplify the RIPC effect.

### 4.7. Limitations

Our study has limitations. First, this is not a randomised controlled trial (RCT). While data in the comparison group were collected prospectively, patients were not randomized into the comparison group or the control group. Instead, we chose propensity score matching to match the comparison cohort to a control cohort, where data were acquired retrospectively. Especially in small trials, this has the theoretical upside of outcomes of matched treated and untreated subjects likely being more similar to one another compared to the outcomes of randomly selected treated and untreated subjects [45]. However, although potential influencing factors are minimized through matching by reducing measured covariates, we cannot rule out that potential unmeasured confounders between the groups might influence the outcome, especially as data were collected differently (prospective vs. retrospective) and during different time frames (2014 to 2015 in control group vs. 2015 to 2016 in RIC group). Nonetheless, during the time frame covered by the study, the standards of data collection, the implantation methods and the post-interventional monitoring did not significantly change. Hence, we estimate the risk of relevant confounding to be low. Second, the study is underpowered and too short to identify a change in mortality. For instance, to obtain a significant reduction of mortality rate of 3% with a statistical power of 80%, a total sample size of 3710 patients would have been necessary. Considering both the limitations to the study design and to the interpretability of mortality, our study is hypothesis generating. For future studies, a randomized controlled trial will be the next logical step. The optimum timing and amount of ischemic conditioning should be further evaluated.

Third, selecting cardiac enzymes to describe the endpoint for myocardial injury is debatable. As an example, cardiac magnetic resonance imaging (CMRI) is more valid for detecting myocardial ischemia and/or edema, thus providing more valuable information on the effect of RIPC on myocardial injury. However, conducting CMRI shortly after a TAVR procedure is not recommended by the prosthesis manufacturer. Cardiac enzymes are commonly used and, furthermore, they are defined by the VARC-2 consortium, thus providing the most easily accessible and comparable data with evidence correlating to mortality and morbidity [26,27,28].

Finally, the efficacy of ischemia under RIPC was controlled by clinical methods only, but with objective criteria. Measurement of serum lactate was proposed as an objective read-out, but was not feasible due its short half-life.

## 5. Conclusions

Our data do not lend support for a beneficial role of RIPC in TAVI patients for cardio- or renoprotection or to improve survival. However, RIPC is a non-invasive, drug-free method without adverse effects. An increase in total ischaemic area or a longer period of RIPC can be additional aspects in further studies.

## Figures and Tables

**Table 1:** Baseline characteristics of RIPC- and matched control-group.

	RIPC-Group (n = 66)	Control-Group (n = 66)	*p*-Value
**Age (years)**	82.2 (4.4)	82.2 (5.6)	0.960
**Gender**			
female	32 (48.5)	31 (47.0)	1.000
male	34 (51.5)	35 (53.0)	
			
**BMI (kg/m^2^)**	28.2 (25.5–31.2)	27.6 (23.8–30.9)	0.171
Body-height (m)	1.77 (0.09)	1.65 (0.09)	0.727
Body-weight (kg)	80 (69–89)	75 (65–86)	0.137
			
**LV-EF (%)**	55 (48–60)	55 (45–60)	0.568
**Aortic stenosis**			
Vmax (cm/sec)	403.6 (94.3) (n = 53)	417.6 (79.4) (n = 59)	0.572
dpmax (mmHg)	72.0 (48.0–85.0) (n = 63)	67.0 (53.5–88.5) (n = 62)	0.907
dpmean (mmHg)	40.0 (28.0–51.0) (n = 63)	40.0 (31.3–53.8) (n = 60)	0.728
			
**CAD (stenosis > 50%)**	33 (50)	30 (46)	0.701
PCI/CABG prior TAVI	26 (39)	24 (36)	0.860
			
**Diabetes mellitus**	26 (39)	27 (41)	1.000
insulin dependent	11 (17)	11 (17)	1.000
			
**Prior medication**			
ACE-Inhibitor/AT1-Blocker	50 (76)	42 (64)	0.215
ß-Blocker	54 (82)	50 (76)	0.541
MRA	6 (9)	6 (9)	1.000
Statins	53 (80)	53 (80)	1.000
Anti-platelets	56 (85)	54 (82)	0.842
**Heart rhythm**			
Atrial flutter/fibrillation	29 (44)	26 (39)	0.720
AV-Block (I-III)	14 (21)	9 (14)	0.359
branch block	25 (38)	17 (26)	0.200
permanent pacemaker	12 (18)	12 (18)	1.000
			
**Euro-II-Score**	4.6 (2.9–7.6)	4.0 (2.7–7.2)	0.728
			
**Valvetyp**			
Medtronic CoreValve	44 (67)	44 (67)	1.000
Edwards Sapien XT/3	22 (33)	22 (33)	
**Prothesis size**	29 (26–29)	26 (26–29)	0.871
23 mm	4 (6)	2 (3)	
26 mm	29 (44)	32 (49)	
29 mm	31 (47)	29 (44)	
31 mm	2 (3)	3 (5)	
**n-dilations**	2 (1–2)	2 (1–2)	1.000
1	18 (27)	16 (24)	
2	40 (61)	43 (65)	
3	6 (9)	7 (11)	
4	2 (3)	0 (0)	
**n-pacing runs**	2 (2–3)	2 (2–3)	0.986
1	40 (61)	35 (53)	
2	18 (27)	25 (38)	
3	6 (9)	6 (9)	
4	2 (3)	0 (0)	
**Procedure duration (min)**	54 (41–71)	52 (42–-65)	0.293
**Contrast medium (ml)**	130 (120–150)	130 (100–153)	0.227
			
**Blood analysis prior TAVI**			
hsTnT (ng/l)	27.0 (14.8–34.3)	23.0 (15.0–51.2)	0.319
Creatinine (µmol/l)	101 (79–134)	98 (81–116)	0.674
GFR (ml/min/KO)	54 (18)	56 (17)	0.473
Urea (mmol/l)	7.1 (5.6–10.8)	7.1 (5.6–9.6)	0.573
Leukocyts (Gpt/l)	7.1 (6.2–8.2)	7.0 (6.2–8.0)	0.928
CRP (mg/l)	3.5 (1.2–7.2)	3.1 (1.5–7.1)	0.682
PCT (µg/l)	0.05 (0.05–0.08) (n = 46)	0.05 (0.05–0.07) (n = 45)	0.554
Interleukin-6 (pg/mL)	5.8 (4.0–8.3) (n = 48)	6.6 (4.5–9.4) (n = 34)	0.803
NTproBNP (ng/l)	2082 (1463–3372) (n = 50)	1948 (1205–4287) (n = 37)	0.679

Values are mean ± SD, median (IQR) or n (%). In case of deviation from n = 66, this is stated [n]. RIPC = remote ischemic preconditioning; BMI = Body-Mass-Index; LV-EF = left ventricular ejection fraction; Vmax = maximum velocity; dpmax = maximum pressure gradient; dpmean = mean pressure gradient; CAD = coronary artery disease; PCI = percutaneous coronary intervention; CABG = coronary-artery bypass grafting; ACE = angiotensin-converting enzyme; AT1 = angiotensin-1-receptor; MRA = mineral-corticoid-antagonist; AV = atrioventricular; hsTnT = high sensitive troponin T; GFR = glomerular filtration rate; CRP = C-reactive-protein; PCT = procalcitonin; NTproBNP = N-terminal pro brain natriuretic peptide.

**Table 2:** Clinical outcomes and complications.

	RIPC-Group (n = 66)	control-Group (n = 66)	*p*-Value
**Death** (from any cause)	7 (11)	9 (14)	0.559
**Stroke/TIA**	2 (3)	0 (0)	0.500
disabling	2 (3)	0 (0)	0.500
non-disabling	0 (0)	0 (0)	1.000
**ppMI**	0 (0)	0 (0)	1.000
**Valve-embolisation**	3 (5)	0 (0)	0.250
**Conversion to surgery**	2 (3)	0 (0)	0.500
**Vascular complications**	6 (9)	4 (6)	0.727
**Major-bleeding complications**	5 (8)	8 (12)	0.581
**New arrhythmia**			
Atrial flutter/fibrillation	6 (9)	5 (8)	1.000
AV-Block I-III	19 (29)	16 (24)	0.711
Branch block	20 (30)	20 (30)	1.000
**New pacemaker**	23 (34)	20 (30)	0.690

No statistically significant differences between RIPC and non-RIPC-group. Values are n (%). RIPC = remote ischemic preconditioning; TIA = transient ischemic attack; ppMI = periprocedural myocardial infarction.

**Table 3:** Kidney-specific parameters for RIPC- and matched control-group.

	RIPC-Group (n = 66)	Control-Group (n = 66)	*p*-Value
**Creatinine-peak** (µmol/L)	99 (77–127)	97 (80–119)	0.684
**Creatinine AUC d5** (µmol/L/d)	442 (351–552)	434 (356–528)	0.752
**GFR decrease**	31 (47)	31 (47)	1.000
**AKIN**	1 (1.5)	4 (6)	0.250
1	1 (1.5)	2 (3)	
2	0 (0)	0 (0)	
3	0 (0)	2 (3)	

No statistically significant differences between RIPC and non-RIPC-group. Values are median (IQR) or n (%). RIPC = remote ischemic preconditioning; AUC = area under the curve; d5 = day 5; GFR = glomerular filtration rate; AKIN = acute kidney injury.

## Supplementary Materials

The following are available online at https://www.mdpi.com/2077-0383/9/1/160/s1. Table S1: Baseline characteristics of RIPC and unmatched control group. Table S2: Standardized difference (d) of matching variables. Table S3: Echocardiographic parameters of RIPC and matched control group.
