# Supplementary material for: Remote Ischemic Preconditioning Neither Improves Survival nor Reduces Myocardial or Kidney Injury in Patients Undergoing Transcatheter Aortic Valve Implantation (TAVI)
Source: J Clin Med. 2020 Jan 7;9(1):160. doi: 10.3390/jcm9010160 (PMC7019611; doi:10.3390/jcm9010160)
Supplement: Supplementary file 1 [file jcm-09-00160-s001.pdf]

Supplementary Tables

Table S1: Baseline characteristics of RIPC and unmatched control group

	RIPC group (n=66)	control group (n=112)	p-Value
**Age** (years)	82 (79-85)	83 (80-86)	0.292
**Gender**			
- female	32 (49)	55 (49)	1.000
- male	34 (52)	57 (51)	
**Body-height** (m)	1.66 (0.09)	1.65 (0.09)	0.520
**Body-weight** (kg)	80 (69.0-89.3)	75 (63.3-85.8)	0.082
**BMI** (kg/m^2)	28.2 (25.5-31.3)	27.5 (23.7-30.5)	0.113
**LV-EF** (%)	55 (48-60)	55 (45-60)	0.793
**Aortic stenosis**			
- Vmax (cm/sec)	403.6 (94.3) [53]	411.1 (79.5) [98]	0.605
- dpmax (mmHg)	72 (48-85) [63]	67 (50.0-82.5) [105]	0.773
- dpmean (mmHg)	40 (28-51) [63]	40 (30.0-51.8) [100]	0.850
**CAD (stenosis > 50%)**	33 (50.0)	53 (47.3)	0.758
**- PCI/CABG prior TAVI**	26 (78.8)	42 (79.2)	0.873
**Diabetes mellitus**	26 (39.4)	49 (43.8)	0.638
**- insulin dependent**	11 (16.7)	23 (20.5)	0.561
**Prior medication**			
- ACE-Inhibitor/ AT1-Blocker	50 (75.8)	75 (67.0)	0.239
- Beta-Blocker	54 (81.8)	87 (77.7)	0.570
- MRA	6 (9.1)	11 (9.8)	1.000
**Heart rhythm**			
- atrial flutter/fibrillation	29 (43.9)	52 (46.4)	0.758
- AV-Block (I-III)	14 (21.2)	16 (14.3)	0.300
- branch block	25 (37.9)	37 (33.0)	0.510
- permanent pacemaker	12 (18.2)	18 (16.1)	0.836
**Euro-II-Score**	4.6 (2.9-7.6)	4.1 (2.8-7.1)	0.672
**Valvetype**			
- Medtronic CoreValve	44 (66.7)	78 (69.6)	0.739
- Edwards Sapien XT/3	22 (33.3)	34 (30.4)	
**Prothesis size**	27.5 (26-29)	26,0 (26-29)	0.978

- 23 mm	4 (6.1)	4 (3.6)	
- 26 mm	29 (43.9)	56 (50.0)	
- 29 mm	31 (47.0)	45 (40.2)	
- 31 mm	2 (3.0)	7 (6.3)	
**n-dilatations**	2 (1-2)	2 (1-2)	0.980
- 1	18 (27.3)	30 (26.8)	
- 2	40 (60.6)	69 (61.6)	
- 3	6 (9.1)	12 (10.7)	
- 4	2 (3.0)	0 (0.0)	
- 5	0 (0.0)	1 (0.9)	
**n-pacing runs**	2 (2-3)	3 (2-3)	0.246
- 2	40 (60,6)	54 (48.2)	
- 3	18 (27.3)	48 (42.9)	
- 4	6 (9.1)	9 (8,0)	
- 5	2 (3.0)	0 (0,0)	
- 6	0 (0.0)	1 (0.9)	
**Procedure duration** (min)	53.5 (41.0-71.3)	50.0 (41-60)	0.285
**Contrast medium volume** (ml)	130 (120-150)	125 (100-150)	0.072
**Blood analysis prior TAVI**			
- hsTnT (ng/l)	27 (14.8-34.3)	24 (16.0-48.8)	0.342
- Creatinine (µmol/l)	101.0 (79.3-133.5)	100.5 (83.3-127.8)	0.735
- GFR (ml/min/KO)	53.8 (17.7)	52.4 (17.3)	0.963
- Urea (mmol/l)	7.1 (5.6-10.8)	7.5 (5.7-10.2)	0.805
- Leukocyts (Gpt/l)	7.1 (6.2-8.2)	7.0 (6.2-7.9)	0.496
- CRP (mg/l)	3.5 (1.2-7.2)	3.1 (1.5-7.8)	0.615
- PCT (µg/l)	0.05 (0.05-0.08) [46]	0.05 (0.05-0.08) [72]	0.909
- Interleukin-6 (pg/ml)	5.8 (4.0-8.3) [48]	7.0 (4.8-9.6) [59]	0.136
- NTproBNP (ng/l)	2082 (1463-3372) [50]	1961 (1085-4139) [64]	0.571

Values are mean ± SD, median (IQR) or n (%). If deviation from n=66, information is given with [n]. RIPC = remote ischemic preconditioning; BMI = Body-Mass-Index; LV-EF = left ventricular ejection fraction; Vmax = maximum velocity; dpmax = maximum pressure gradient; dpmean = mean pressure gradient; CAD = coronary artery disease; PCI = percutaneous coronary intervention; CABG = coronary-artery bypass grafting; ACE = angiotensin-converting enzyme; AT1 = angiotensin-1-receptor; MRA = mineral-corticoid-antagonist; AV = atrioventricular; hsTnT = high sensitive troponin T; GFR = glomerular filtration rate; CRP = C-reactive-protein; PCT = procalcitonin; NTproBNP = N-terminal pro brain natriuretic peptide

Table S2: Standardized difference (d) of matching variables

Parameter	d	
	before PSM	after PSM
Gender	0.01	0.03
LV-EF	0.05	0.01
CAD (stenosis > 50%)	0.05	0.09
Valvetyp	0.06	0.00
n-Pacing runs	0.11	0.01
Contrast amount	0.33	0.12
Creatinine prior TAVI	0.15	0.01

PSM = propensity score matching; LV-EF = left ventricular ejection fraction; CAD = coronary artery disease; TAVI = transcatheter aortic valve implantatin

Table S3: Echocardiografic parameters of RIPC and matched control group

	RIPC group (n=66)	Control group (n=66)	p-Value
**pre TAVI**			
LV-EF (%)	55 (47.9-60.0)	55 (54-60)	0.568
Vmax (cm/sec)	403.6 (94.3) [53]	417.6 (79.4) [59]	0.439
dpmax (mmHg)	67.0 (29.5) [63]	71.1 (25.2) [62]	0.907
dpmean (mmHg)	40 (28-51) [63]	40 (31.3-53.8) [60]	0.728
**post TAVI**			
LV-EF (%)	60 (50-60) [65]	55 (50-60) [63]	0.552
Vmax (cm/sec)	200 (153.0-232.5)	190 (165-225) [63]	0.940
dpmax (mmHg)	16 (10.0-22.5) [65]	15 (11-20) [61]	0.789
dpmean (mmHg)	8 (5-11) [65]	7 (6-10) [61]	0.658
Aortic regurgitation (°)	[65]	[62]	
- 0	26 (40.0)	19 (30.6)	0.527
- 1	36 (55.4)	39 (62.9)	
- 2	1 (1.5)	2 (3.2)	
- 3	2 (3.1)	2 (3.2)	
**follow up 1**			
LV-EF (%)	60 (53-60) [47]	60 (50-60) [44]	0.682
Vmax (cm/sec)	195.0 (38.7) [46]	185.9 (43.3) [43]	0.921
dpmax (mmHg)	15.5 (11.8-19.0) [46]	13 (11-18) [44]	0.749
dpmean (mmHg)	8 (6-11) [46]	7 (5-10) [42]	0.744
Aortic regurgitation (°)	[48]	[45]	0.240
- 0	19 (39.6)	13 (28.9)	

- 1	29 (60.4)	30 (66.7)	
- 2	0 (0)	0 (0)	
- 3	0 (0)	2 (4.4)	
**follow up 2**			
LV-EF (%)	60 (55-60) [38]	55 (55-60) [40]	0.780
Vmax (cm/sec)	197.1 (48.0) [34]	192.8 (39.0) [37]	0.330
dpmax (mmHg)	15 (11.5-21.0) [37]	14 (11.0-18.3) [38]	0.457
dpmean (mmHg)	8 (5-10) [35]	8 (5.5-10.0) [37]	0.721
Aortic regurgitation (°)	[36]	[40]	
- 0	15 (41.7)	13 (32.5)	0.460
- 1	21 (58.3)	22 (55.0)	
- 2	0 (0)	4 (10.0)	
- 3	0 (0)	1 (2.5)	

Values are mean ± SD, median (IQR) or n (%). If deviation from n=66, information is given with [n]. RIPC = remote ischemic preconditioning; LV-EF = left ventricular ejection fraction; Vmax = maximum velocity; dpmax = maximum pressure gradient; dpmean = mean pressure gradient
